# Needs and Requirements of Modern Biobanks on the Example of Dystonia Syndromes

**DOI:** 10.3389/fneur.2017.00009

**Published:** 2017-01-30

**Authors:** Ebba Lohmann, Thomas Gasser, Kathrin Grundmann

**Affiliations:** ^1^Department of Neurodegenerative Diseases, Hertie Institute for Clinical Brain Research, University of Tübingen, Tübingen, Germany; ^2^DZNE, German Center for Neurodegenerative Diseases, Tübingen, Germany; ^3^Istanbul Faculty of Medicine, Department of Neurology, Behavioral Neurology and Movement Disorders Unit, Istanbul University, Istanbul, Turkey; ^4^Department of Medical Genetics and Applied Genomics, University of Tübingen, Tübingen, Germany

**Keywords:** biobank, rare disease, dystonia, genes, Euro-dystonia

## Abstract

Dystonia belongs to a group of rare diseases (RDs) characterized by etiologic heterogeneity, affection often in childhood, severe and variable clinical manifestation. The burden of this disease is aggravated by the lack of effective and specific treatment. In the field of dystonia as in other RDs the number of available biospecimens is, in general, limited. Here, we report a new approach to collect clinical and genetic data in biospecimens maintained collaboratively by researchers and their associated institutions in a decentralized system. Allowing researchers to have access to significant numbers of samples and corresponding clinical data, biobanking in dystonia might not only provide a powerful tool in the identification of disease genes but also the classification of variants detected in known genes with respect to their clinical relevance. Growing data in genetics due to the technical progress demand for well-annotated and well-managed biobanks, which in near future hold even the potential for biomarker research and generating medical treatment based on clinical and genetic data currently summarized as “personalized medicine.”

## Introduction

Rare diseases (RDs), also called orphan diseases, of which there are approximately 5.000–8.000, are diseases that affect a small number of people compared to the general population, and specific issues are raised in relation to their rarity. They are often genetic in origin (1, 2). Specific challenges for health care and for research are the rarity and the heterogeneity of RDs. These also affect the development of therapies and their marketing. As a result, many patients with RDs do not receive a timely and accurate diagnosis (3, 4), have to consult numerous doctors to obtain a final diagnosis, and only few receive tailored treatment influencing survival and/or quality of life. It is because of these specific challenges that the EU Public Health Programme 2008–2013 has made RDs a priority area for action. A large number of RD-focused projects have been selected for under the Sixth and Seventh Framework Research Programmes (5). More recently, a proportion of Horizon 2020, one of the largest EU Research and Innovation programmes with a budget of almost 80 million Euro, is specifically aimed at RD initiatives (6). Experts now hope that on the basis of their genetic knowledge and pathway definition, they will be able to develop a new concept, often called “precision medicine.” This should change our view on how to apply therapeutic targets. Collecting clinical and genetic data and also biospecimens (biobanks) will become more important in the diagnosis and therapy development for RDs.

To look for an example of a RD, we should look at dystonia. Dystonia is a hyperkinetic movement disorder and is characterized by sustained muscle contractions with repetitive movements and also abnormal postures. It is difficult to establish specific information on the prevalence of dystonia because of the lack of a representative number of population-based studies. However, based on the current data, the overall prevalence of “primary” dystonia, an umbrella term describing a number of familial and sporadic forms of the disease, is calculated at 16.43 per 100,000 (95% confidence interval: 12.09–22.32) (7).

In this article, our aim is to review the current situation and future direction of RD biobanks by looking at examples of dystonic syndromes and discussing the research and development arising from the use of biospecimens, so as to improve the disease management.

## Definition of the Rare Diseases Dystonia

Rare diseases can manifest at any stage in life, though generalized or severe forms often start in early childhood. Common RD characteristics are genetic, severe, disabling, non-preventable, sometimes fatal, progressive, and having no specific effective treatments (8, 9). Recently, the Australian rare disease community proposed a definition for RD as being “a life-threatening or chronically debilitating disease which is statistically rare, (with an estimated prevalence of less than 1 in 2,000 or of similarly low prevalence) and has a high level of complexity such that special combined efforts are needed to address the disorder or condition” (10).

Dystonia, a good example of a RD, meets this definition, even if it does not describe a specific disease or pathomechanism. Dystonia is a general term for a large group of movement disorders. These may vary in their symptoms, their causes, their progression, and their treatments (Table 1). Dystonia generally shows sustained or intermittent muscle contractions. These can cause abnormal and often repetitive movements and postures. Dystonic movements will often be patterned and twisting, may be tremulous, affecting the neck, the torso, the limbs, the eyes, the face, the vocal chords, and even a combination of these muscle groups. Dystonia can be initiated and worsened by voluntary action and is associated with overflow muscle activation (11). Dystonic postures may cause varying degrees of pain and disability. These can range from occasional and mild symptoms to severe, debilitating symptoms, significantly affecting a person’s quality of life. Dystonia can become progressively worse but remains unchanged in some cases. In rare cases, it has been known to spontaneously remit. Treatment will depend on several factors, e.g., specific subtype and can include medication, botulinum toxin injections, physical therapy, or even surgery.

**Table 1:** **Examples for dystonic syndromes**.

Blepharospasm
Writer’s cramp
Spasmodic dysphonia
Cervical dystonia
Oromandibular dystonia
Meige syndrome
Myoclonic dystonia
Generalized dystonia
Rapid-onset dystonia-parkinsonism
Paroxysmal kinesigenic dystonia
Paroxysmal dystonia choreoathetosis
DOPA-responsive dystonia
Tardive dystonia

Although various elements can contribute to the development of dystonia, in many cases, the exact, underlying causes are unknown. Despite the etiological heterogeneity, the thematic similarities apparent across the dystonic syndrome natural history spectra do enable coordinated approaches. It has been shown, for example, that genetic factors play an important role in the development of dystonic syndromes (12). However, obtaining a genetic diagnosis for dystonia is often difficult because of the lack of appropriate diagnostic tests. A diagnosis is important for the affected patient and his family, though, even when available, the costs of validated tests are not always covered by insurance companies. This is despite the fact that molecular characterization of the sample being critical to a correct diagnosis. This would provide patients with specific genetic counseling, subsequent better care and follow-up. Another important aspect of the genetic diagnosis is its use in research, e.g., giving thought-provoking impulses in order to develop models of the pathomechanism involved (13). Samples, however, are currently only available through highly specialized centers and are stored in a particular system for a population because of the specific research involved.

## The Discipline of Biobanking

Recently, the paradigm in medicine of “reactive approaches” centered on disease therapy is moving to a more personalized, prognostic, preventative, and contributing approach, focusing on the conservation of health (14). This development is promoted by advances acquired from sequencing of the whole human genome and the rapid development in bioinformatics and analytical laboratory technologies (15). Biobanks, as repositories for the storage of this biological material and its corresponding data, could become important tools and instruments in driving this change in the way health care is delivered.

Instead of the subdivision of complex biological phenomena, interdisciplinary efforts are actually made to address the substantial complexities of human biology and medicine. In these young scientific disciplines, advanced mathematical and statistics strategies support the research into the interactions of individual biological elements. These systems not only can retrospectively analyze biological parameters but can also model *in silico* different interactions. Systems biology research combines “wet laboratory” experimentation with “dry laboratory” predictions of the biological processes (14, 15). All these developments, which took off approximately a decade ago, have helped establish organized biobanking (16, 17). The development of guidelines for the standardization of workflow methods that ensure sample quality and stability is ongoing (18). When it comes to the future of human medicine and particularly to that of RDs such as dystonia, biobanking may hold the key, and the standardization of biobanking workflows will ensure a promising future.

## The Field of Biobanking

Biobanks will typically:

– collect and store biological materials. These should be labeled not only with medical data but also with epidemiological data (e.g., environmental exposure, lifestyle, and occupational information);
– not be static “projects,” as biological materials and data are generally collected on a continuous or long-term basis;
– be associated with current (defined) or future (not yet specified) research projects at the time of biospecimen collection;
– apply encoding or be anonymous to ensure donor privacy though under certain circumstances will allow the participants to remain identifiable in order to provide clinically relevant information back to the donor;
– include established governance structures (e.g., ethics review committees) and procedures (e.g., consent) that serve to protect donors’ rights and stakeholder interests.

The field of biobanking is, generally speaking, very heterogeneous (16, 17). Although it is difficult to list all distinguishing characteristics of biobanks exhaustively, there are some that can be used to characterize different types of biobanks. These are size, research design, the types of biological samples collected, the method of sample collection, processing and storage, and the disease/research focus. These characteristics will influence the scope of biobank activities, such as the recruitment of donors, the consent procedures, the scale of IT support needed, the structure of its administration, and the potential for commercial usage. In the past, the terminology describing organized collections in medicine has not been consistent. Various terms have been in use to refer to activities involving biobanks/biobanking, such as “human genetic research databases (HGRDs),” “population genetic databases,” “biorepositories,” or “tissue banks.” However, the term “biobank” has now been established.

Multiple biobank formats can be found within the context of medical research. These can be differentiated, based on their design and scientific aim (Table 2). However, all biobank formats are linked in some form and, to a certain degree, represent a continuum within the infrastructure supporting all steps in the biomedical research “pipeline” (16, 17).

**Table 2:** **Biobank designs**.

Biobank	Characteristics
Population-based	Find the biomarkers for disease susceptibility within a specific population through prospective molecular epidemiology research. Recruitment of healthy donors, typical of a region, country, or specific ethnicity. DNA isolated from venous blood is the most commonly stored biospecimen. Associated data comprising medical history, physical measurements, and epidemiological data (e.g., lifestyle habits, socioeconomic status).

Disease-orientated	Collection of biological materials, collected within the context of clinical care. Patients will only provide biological material and will eventually provide more samples at follow-up visits during the course of their treatment. A number of different disease-oriented biobank subtypes exist.

Case–control	A selection of matched (age and sex as a minimum) individuals presenting a given disease. These will be matched with compatible healthy controls. Epidemiological case–control studies can be used as biobanks. Population-based biobanks can provide case–control.

Tissue banks	Extremely diverse collections of tissue specimens. Usually invasive sampling followed by cryopreservation. Detailed information on the nature of the underlying disease. Specific form of tissue banks, e.g., formalin-fixed paraffin-embedded specimen collections.

Biobanking within the context of clinical trials	Performed by research organizations and/or investigator-driven clinical trials. Associated with complex clinical and laboratory monitoring data. Examine samples (e.g., blood, urine), which can in turn be integrated into a biobank and used for research. Aim is to identify disease/trial-associated biomarkers.

Other specific biobanking formats	Specific methods are necessary requiring deep experience (e.g., cell cultures of pluripotent cells). Specific research goals (Guthrie cards). Commercial interest with the regard to future regenerative therapies (cord blood).

## Biobanks for Dystonia

It is important to access biological materials for scientific research in all medical fields and particularly for research on RDs such as dystonic syndromes, though it is difficult to obtain high quality samples and related clinical data. Biobanks play a major role in providing such materials and data to the scientific community.

Nevertheless, up to date, it is difficult to determine the exact number, nature, and quality of samples from patients with dystonic syndrome included in the various biobanks.

An example of a large initiative that is currently running for the study of dystonia is the “Dystonia Coalition” funded by the National Institutes of Health, USA and aims to establish a centralized biobank of samples for the study of dystonic syndromes. More than 40 participating clinical centers are distributed throughout North America and Europe.^1^

The European counterpart is the Europe Dystonia (ED) group, a group of researchers who have developed a web-based integration of their research activities on the genetics of dystonia.^2^ The ED research network presently consists of partners from 16 European countries and Israel. The ED registry and biobank is a collaborative network project supported by all members of the ED research network. It consists of a web-based registry for clinical and genetic data, hosted by the University of Tübingen, and a decentralized biorepository at different research sites.

The structure and the organization of the ED registry fulfill all the requirements of a modern and sustainable biobank system, and in the following sections, the biobank will be explained in more detail.

### European Dystonia Registry and Biorepository

#### Organization

The network includes two types of participating centers, depending on local resources and interests: “Collection only” centers and “Collection-DNA storage” centers. The “Collection only” centers include all Euro-dystonia centers willing to collect clinical data, enter them into the Clinical Dystonia Registry in a standardized way, and draw blood for DNA extraction on patients with dystonia and their consenting relatives, but with no facilities for DNA extraction or storage. In comparison, the “Collection-DNA storage” centers perform all tasks of the “Collection only” center, but, in addition, extract and store samples, grow cells, and make the biospecimen available for research. This combination of centralized and decentralized biobanking combines in a perfect way the strengths of both models: high quality in the control of biospecimens, data management, and operation *via* standardized and harmonized methods of samples extraction and storage between the collecting centers. This combination of both systems is faster, more likely to preserve as many analytes as possible, is suitable for very complex protocols, and leaves space for flexibility and innovation without incurring high setup or transport costs.

#### Data Handling

Protection of personal data is ensured according to local legal regulations. DNA, personal data, and genetic data are only documented, stored, and analyzed anonymously. Identification of samples and patients may be necessary in order to obtain additional clinical information or to inform the patient that clinically relevant results have been obtained. Identification lists are only available to an authorized clinical investigator at the participating center. Local ethics boards have to approve all procedures. It is important to correlate data and biospecimens from different biobanks, this being crucial in accelerating the pace of translational research. A process of harmonization is crucial in order to share the best practices and procedures in biobanks. This more flexible approach aims at ensuring the effective interchange of valid information and samples (19).

All patients and relatives must be fully informed about the study, in particular, about the voluntary nature of participation and the possibility of withdrawing and having the sample destroyed, at any time. Depending on the local procedure based on the local ethical approval, patients and relatives are also informed that no individual test results will be given, but that information about research results in general can be obtained from the attending physician at the ED center. If clinical genetic testing should become available, based on results of the scientific studies, and the patient has chosen on the consent form to be informed, a formal testing procedure may be suggested to the patient and its relatives according to general guidelines for genetic testing at a certified laboratory. The general procedure in reporting the results of the findings from the use of biospecimens is publishing these for the benefit of the scientific community. Findings that fall outside the research objectives and that have potential health or clinical significance are termed “incidental finding” (20). A debate has recently emerged over the need to return incidental findings, and policies are rapidly being developed to cover actual and future obligations (21). Nevertheless, a harmonized and ethically defensible plan for the return of incidental findings is not yet available, and the final decision is taken according to the evaluation of the responsible clinician.

#### Ownership

Ownership of (personal) data is a very important issue. Since there are obviously numerous stakeholders in a biobank—the donors, the investigators, the funding agencies, the institution housing the bio-samples, and the ethics review committee—it has been proposed that the institution of the biobank should hold “custodianship” for the use of the resource, and that, as custodian of the samples, should carry numerous responsibilities (22). However, the members of the *European Dystonia Registry and Biorepository* concluded that clinical data and biomaterials remain in the possession of the donor unless national legal regulations determine differently.

#### Data and Biomaterial Access

All data and DNA-samples are accessible at all times available with no restrictions for the participating centers. Pseudonymized data and DNA-samples are made available to other researchers upon request according to the procedure detailed below. A steering committee has been elected by the majority of the votes of all participating centers of the ED and deals with questions of distribution of the DNA resources for scientific projects on the basis of the procedure outlined below.

#### Use of Samples

Samples can also be made available to research groups within and outside the ED collaboration. For example, in the case of dystonia, the samples will be used for the study of possible genetic factors in the development and course of the disease, and a research proposal will be submitted to the ED steering committee. The committee then submits a recommendation to the contributing center based on the scientific merits of the proposal. The steering committee will ensure that all laboratories receiving samples from ED agree in writing that

- samples will be used only for the project applied for;
- samples will not be passed on to other laboratories without express permission; and
- an appropriate publication policy will be guaranteed.

However, the final decision on the use of samples will remain with the local contributing scientists. Contributors will be informed about all study proposals and sample allocations. In some cases, fees can be charged for the maintenance of the biobank.

In addressing the social and ethical challenges of biobanking (23), proper governance is vital to the success of biobanking initiatives: it is crucial in ensuring the safety and protection of participants, in preserving public support and financing, and in safeguarding the availability of biospecimens for research (24, 25). This is especially applicable to RD biobanking efforts because of the rarity and diversity of biomaterials and the role played by patients and patient organizations (13).

#### Scientific Publications

A major challenge is in how to honor and recognize the effort and the expertise involved in establishing a biobank. Until recently, there was a direct link between the researcher who set up a data set and the background for many publications. This relationship in biobanking, between the custodian and the biobank, is very different, as the biobank has been established as a resource open to others. The custodian of the biobank may or may not, carry out research on material from the biobank. Therefore, the traditional ways of acknowledging researchers through their publications, which may further their careers, become difficult for the custodians of biobanks. Many journals require that data production should be acknowledged, but how this is done is largely left up to individuals who follow the norms that exist in their particular discipline (26).

If results of scientific studies based on the ED collaboration are published, according to good scientific practice, authorship is based on individual contributions. In accordance with the published guidelines of scientific journals, contribution of a limited number of DNA-samples alone is not necessarily sufficient to justify coauthorship. However, regarding all papers based on the use of ED samples, it is proposed to include the following sentence: “and members of Euro-Dystonia Network,” with at least one representative of each participating center, which has provided DNA, being listed in an appendix. An accepted significant scientific contribution, justifying coauthorship, would be the contribution of a substantial number of samples with thorough clinical data documentation. This contribution is admittedly hard to evaluate and would need to be assessed case by case.

## Benefits and Challenges of Biobanks for Dystonia

Biobanks for dystonia support the adaptation of laboratory research into clinical applications, the defined goal being to harmonize diagnostic or therapeutic tools for the disease (13, 27). In dystonia, with valuable but limited amounts of biomaterial scattered over a large area, biobanks are a major resource. The purpose of the biobank very much determines its benefits. The purposes vary greatly from generating revenue, supporting scientific discoveries and the understanding the causes of the disease, genetic testing, assessing drug efficacy and treatment, identifying new genes and biomarkers, clinical trials, education, or personalized therapy. Biospecimens held in biobanks have helped researchers and clinicians in their understanding of the mechanism and underlying causes, particularly of RDs, and have also helped with gene identification and the development of diagnostic and therapeutic biomarkers. New genes and new gene mutations have been discovered, thanks to collections of DNA (28–31), as has the development of new diagnostic criteria (32), and the defining of genotype–phenotype correlations (33, 34). Analysis of serum and of plasma has facilitated the identification of new biomarkers (35, 36) and protein profiles (37). Other biospecimens, such as mRNA, stem cells, and tissue, have helped to collect functional data in order to identify other pathways and new therapies to be applied to RDs (38–40).

Three major challenges need to be met in order to increase the effectiveness of biobanks for dystonia: maximizing access for the international scientific community to rare biological samples stored in biobanks across the globe, promoting networking among such biobanks in order to share and harmonize quality standards and procedures and allowing collaboration with dystonia registries and databases, and finally adopting an efficient management model compliant with legal and ethical issues, ensuring biobank sustainability.

Historically, research in dystonia has been highly fragmentary, according to data type, research institution, or dystonic syndrome. There is a limited number of biospecimens for most types of dystonic syndromes, and it may be difficult or impossible to increase the sample number in a short time frame, making these samples extremely precious. Additionally, to achieve usefulness, the quality of the biomaterials and of the associated information is of primary importance (27). Some research biobanks held in laboratories with non-interoperable databases can make it almost impossible to connect genetic data with detailed clinical information or biospecimen availability.

To achieve this, it is important to increase the awareness of dystonia, to increase the number of dystonia registries and biobanks accessible to concerned patients and families, and to expand the availability of biospecimens from these patients. In this context, it is notable that the attitudes toward biobanks are still far from being settled in many countries. In general, there is a cluster of Northern European countries where the prospect of biobank research is greeted enthusiastically, whereas the general public of many Central and Southern European countries is generally more reserved about biobank research, providing tissue samples and granting broad consent for research (41). This reserve has implications for recruitment and also for the running and governance of biobanks. Another particularity with decentralized international biobank network for dystonia is the fact that ethical guidelines and the patient’s attitude toward biobanks often differ widely between different countries (42). At the outset, all ethical considerations should be in line with national guidelines of the patient’s country and where the biomaterial has been obtained. However, there exists no global consensus regarding the sharing of biomaterial and data. Eventually, consent will move on to becoming ongoing and dynamic; participants will be able to engage as much as they wish and to change their consent choices over time (20).

## The Genetic Aspect

Dystonia is also a very challenging disease from a genetic point of view. First, a number of clearly genetic conditions are still undefined. Second, penetrance of mutations in known genes is low. Third, we observe a broad phenotypic variability in the majority of mutations in known genes. In conclusion, the predictive value of mutations in known genes is low. Even if the mutation is known, we cannot tell whether the person will be affected, and if affected, how seriously. Furthermore, in some countries, prenatal diagnosis is not allowed for mutations with reduced penetrance and uncertain clinical prognosis.

On the other hand, with the introduction of the next-generation sequencing, sequencing of the whole genome in an affordable time frame is now possible. Accordingly, next-generation sequencing increases the pace of discovery of novel genes, even in RDs, tremendously (43). As genetic sequencing becomes cheaper and more accessible, we are about to face an increase of genetic tests in dystonia and subsequently, an increase in unclear variants. In particular, analysis of genes in RDs leads to results associated with extremely limited information about penetrance, phenotype, and prevalence. Moreover, Bell and colleagues recently observed that a high proportion of disease mutations (27%) are incorrectly or incompletely annotated (44). Thus, for many recessive orphan diseases, standard open accessible databases such as HGMD, dbSNP, or OMIM provide insufficient information (44). Identification of potentially underlying genetic causes by means of comprehensive analysis of gene sequences is technically feasible though the clinical interpretation of the functional importance or of the pathogenicity of variants will be challenging for numerous genetic diseases and would require the establishment of an authoritative disease mutation database. Otherwise, the clinical usefulness of comprehensive carrier testing will be limited. The benefit of a transnationally organized biobank, including the molecular characteristic and also results of clinical and paraclinical examinations of the patients and their family members, is based on greater flexibility, particularly regarding the genetic approach. This is mainly because they can support a variety of studies, including cross-sectional studies of genotype–phenotype correlations, case–control studies using a biobank for cases and/or controls, and cohort studies using baseline and follow-up data in a biobank to link genetic variation with health outcomes (45).

## Future Perspectives

Bringing dystonia biobanks to the attention of scientists, clinicians, and patients will require further efforts. These will also be necessary in order to closely link and receive information from specialized diagnostic centers and disease experts, for further qualitative and quantitative progress. It is important that clinical staff should have a clear understanding of the added value of participating in the biobank network. They should be encouraged to collect samples and to update relevant databases. These efforts are a generous contribution to global health research, but they are also a direct path to advancing personal scientific and medical activities (46). It is important to establish an accreditation and evaluation system so as to acknowledge biobanks providing high quality samples and to reward and recognize the scientists who establish and maintain biobanks.

Here, the promotion of collaboration between biobanks and patient associations will not only help collect more samples and associated data and furnish them to researchers but could also help address the ethical and legal challenges, thanks to the underlying agreements based on solidarity.

Sustainability is another important issue facing dystonia biobanking. The pharmaceutical industry, clearly, has little interest in funding small biobanks that contain and exchange small numbers of samples, making specific funding for dystonia biobanks essential. An approach to this problem could be the implementation of a business model in which both cost and revenue are examined. However, the commercialization of the biobank biomaterial and/or clinical data is not always a feasible option. National or institutional regulations, ethical guidelines, or patients refusing informed consent, all prevent any commercialization and limit the willingness of the pharmaceutical industry to get involved.

In order to unify and simplify the practice of biobanking across multiple institutions and different countries, it would be essential to harmonize the legal and regulatory frameworks that apply. Additionally, interoperability and harmonization of dystonia patient registries and dystonia biobanks are critical for linking data, together with time-efficient procedures adapted to the clinical workflow promoting clinical engagement and enhancing diagnostic and therapy development for dystonic syndromes.

## Conclusion

The challenge for the future of biobanking in RDs as dystonia is how to develop governance that encourages a sustainable international biobanking infrastructure. Presently, the regulatory systems are not harmonized, thus hindering straightforward sharing of samples and data in an ethical and legally compliant manner across national borders. This does not aid cutting-edge research across borders in the most efficient and economical manner. The governance structure for medical research needs to move from being designed around “one-researcher, one-project, one-jurisdiction” model to enable the flow of samples and data between biobanks as part of regional/global networks for research. One way to achieve this is to use information technology to develop e-governance systems that can increase the transparency of the research done and augment existing expert committee review and national systems of oversight. This will enable dystonia biobanks to become component parts of the health-care structure and a tool to enhance a personalized medicine approach to health care, respecting the participants’ fundamental rights and researchers’ needs. All future developments should take account of other efforts undertaken elsewhere in the world.

## Author Contributions

EL: analysis and interpretation of data and drafting the manuscript. KG: analysis and interpretation of data. TG: critical revision of the manuscript for important intellectual content.

## Conflict of Interest Statement

The authors declare that declare that the research was conducted in the absence of any commercial or financial relationships that could be construed as a potential conflict of interest.

## Footnotes

^1^http://www.rarediseasesnetwork.org/dystonia.

^2^http://dystonia-europe.org.

## References

[1] European Organisation for Rare Diseases. Rare Diseases: Understanding This Public Health Priority. Paris: EURORDIS (2005).

[2] Aymé S Rodwell C Report on the State of the Art of Rare Disease Activities in Europe of the European Union Committee of Experts on Rare Diseases – Part I: Overview of Rare Disease Activities in Europe and Key Developments in 2010. (2011). Available from: http://www.eucerd.eu/upload/file/Reports/2011ReportStateofArtRDActivities.pdf

[3] Bertram KL Williams DR. Delays to the diagnosis of cervical dystonia. J Clin Neurosci (2016) 25:62–4. 10.1016/j.jocn.2015.05.054 26601813

[4] Macerollo A Superbo M Gigante AF Livrea P Defazio G. Diagnostic delay in adult-onset dystonia: data from an Italian movement disorder center. J Clin Neurosci (2015) 22(3):608–10. 10.1016/j.jocn.2014.09.014 25577433

[5] Innovation Directorate-General for Research and Innovation. Rare Diseases –How Europe IS Meeting the Challenges. Luxembourg (2013).

[6] Almasy L Bressman S de L Risch N. Ethnic variation in the clinical expression of idiopathic torsion dystonia. Mov Disord (1997) 12(5):715–21. 10.1002/mds.870120515 9380054

[7] Steeves TD Day L Dykeman J Jette N Pringsheim T. The prevalence of primary dystonia: a systematic review and meta-analysis. Mov Disord (2012) 27(14):1789–96. 10.1002/mds.25244 23114997

[8] Directorate-General for Research and Innovation. Rare Diseases – How Europe Is Meeting the Challenges. Luxembourg: Publications Office of the European Union (2013).

[9] Jaffe A Zurynski Y Beville L Elliott E Call for a national plan for rare diseases. J Paediatr Child Health (2009) 46:2–4. 10.1111/j.1440-1754.2009.01608.x 19943870

[10] Dawkins H Molster C Youngs L O’Leary P Awakening Australia to Rare Diseases: Symposium report and preliminary outcomes. Orphanet J Rare Dis. (2011) 6:57 10.1186/1750-1172-6-57 21849083 PMC3170182

[11] Albanese A Bhatia K Bressman SB DeLong MR Fahn S Fung VSC Phenomenology and classification of dystonia: a consensus update. Mov Disord (2013) 28(7):863–73. 10.1002/mds.25475 23649720 PMC3729880

[12] Klein C Marras C Münchau A Dystonia overview. In: Pagon RA Adam MP Ardinger HH, editors. GeneReviews^®^. Seattle, WA: University of Washington (2003). p. 1993–2017. Available from: https://www.ncbi.nlm.nih.gov/books/NBK1155/20301334

[13] Lochmuller H Schneiderat P. Biobanking in rare disorders. Adv Exp Med Biol (2010) 686:105–13. 10.1007/978-90-481-9485-8_7 20824442

[14] Loscalzo J Barabasi AL Systems biology and the future of medicine. Wiley Interdiscip Rev Syst Biol Med (2011) 3(6):619–27. 10.1002/wsbm.144 21928407 PMC3188693

[15] Offit K. Personalized medicine: new genomics, old lessons. Hum Genet (2011) 130(1):3–14. 10.1007/s00439-011-1028-3 21706342 PMC3128266

[16] Asslaber M Zatloukal K. Biobanks: transnational, European and global networks. Brief Funct Genomic Proteomic (2007) 6(3):193–201. 10.1093/bfgp/elm023 17916592

[17] Riegman PH Morente MM Betsou F de Blasio P Geary P Marble Arch International Working Group on Biobanking for Biomedical R Biobanking for better healthcare. Mol Oncol (2008) 2(3):213–22. 10.1016/j.molonc.2008.07.004 19383342 PMC5527804

[18] Malm J Fehniger TE Danmyr P Vegvari A Welinder C Lindberg H Developments in biobanking workflow standardization providing sample integrity and stability. J Proteomics (2013) 95:38–45. 10.1016/j.jprot.2013.06.035 23856607

[19] Fortier I Doiron D Burton P Raina P Invited commentary: consolidating data harmonization – how to obtain quality and applicability? Am J Epidemiol (2011) 174(3):261–4; author reply 5–6. 10.1093/aje/kwr194 21749975

[20] Knoppers BM Zawati MH Kirby ES. Sampling populations of humans across the world: ELSI issues. Annu Rev Genomics Hum Genet (2012) 13:395–413. 10.1146/annurev-genom-090711-163834 22404491

[21] Knoppers BM Deschenes M Zawati MH Tasse AM. Population studies: return of research results and incidental findings Policy Statement. Eur J Hum Genet (2013) 21(3):245–7. 10.1038/ejhg.2012.152 22781095 PMC3573193

[22] Vaz M Vaz M Srinivasan K. Ethical challenges in biobanking: moving the agenda forward in India. Indian J Med Ethics (2014) 11(2):79–88. 24727618 10.20529/IJME.2014.022

[23] O’Doherty KC Hawkins A. Structuring public engagement for effective input in policy development on human tissue biobanking. Public Health Genomics (2010) 13(4):197–206. 10.1159/000279621 20395688 PMC2874727

[24] European Commission, Directorate-General for Research and Innovation. Biobanks for Europe – A Challenge for Governance. Luxembourg (2012).

[25] Gottweis H Lauss G. Biobank governance: heterogeneous modes of ordering and democratization. J Community Genet (2012) 3(2):61–72. 10.1007/s12687-011-0070-0 22147279 PMC3312942

[26] Kaye J Heeney C Hawkins N de Vries J Boddington P Data sharing in genomics – re-shaping scientific practice. Nat Rev Genet (2009) 10(5):331–5. 10.1038/nrg2573 19308065 PMC2672783

[27] Lochmuller H Ayme S Pampinella F Melegh B Kuhn KA Antonarakis SE The role of biobanking in rare diseases: European consensus expert group report. Biopreserv Biobank (2009) 7(3):155–6. 10.1089/bio.2010.7302 24835882

[28] Magri F Del Bo R D’Angelo MG Govoni A Ghezzi S Gandossini S Clinical and molecular characterization of a cohort of patients with novel nucleotide alterations of the dystrophin gene detected by direct sequencing. BMC Med Genet (2011) 12:37. 10.1186/1471-2350-12-37 21396098 PMC3061890

[29] Mencacci NE Rubio-Agusti I Zdebik A Asmus F Ludtmann MH Ryten M A missense mutation in KCTD17 causes autosomal dominant myoclonus-dystonia. Am J Hum Genet (2015) 96(6):938–47. 10.1016/j.ajhg.2015.04.008 25983243 PMC4457957

[30] Zimprich A Grabowski M Asmus F Naumann M Berg D Bertram M Mutations in the gene encoding epsilon-sarcoglycan cause myoclonus-dystonia syndrome. Nat Genet (2001) 29(1):66–9. 10.1038/ng709 11528394

[31] Doss S Lohmann K Seibler P Arns B Klopstock T Zuhlke C Recessive dystonia-ataxia syndrome in a Turkish family caused by a COX20 (FAM36A) mutation. J Neurol (2014) 261(1):207–12. 10.1007/s00415-013-7177-7 24202787

[32] Scionti I Greco F Ricci G Govi M Arashiro P Vercelli L Large-scale population analysis challenges the current criteria for the molecular diagnosis of fascioscapulohumeral muscular dystrophy. Am J Hum Genet (2012) 90(4):628–35. 10.1016/j.ajhg.2012.02.019 22482803 PMC3322229

[33] Anichini A Fanin M Vianey-Saban C Cassandrini D Fiorillo C Bruno C Genotype-phenotype correlations in a large series of patients with muscle type CPT II deficiency. Neurol Res (2011) 33(1):24–32. 10.1179/016164110X12767786356390 20810031

[34] Terry SF Terry PF Rauen KA Uitto J Bercovitch LG. Advocacy groups as research organizations: the PXE International example. Nat Rev Genet (2007) 8(2):157–64. 10.1038/nrg1991 17230202

[35] Quero C Colome N Rodriguez C Eichhorn P Posada de la Paz M Gelpi E Proteomics of toxic oil syndrome in humans: phenotype distribution in a population of patients. Chem Biol Interact (2011) 192(1–2):129–35. 10.1016/j.cbi.2010.11.001 21075095

[36] Ramirez RL Qian J Santambrogio P Levi S Koeppen AH. Relation of cytosolic iron excess to cardiomyopathy of Friedreich’s ataxia. Am J Cardiol (2012) 110(12):1820–7. 10.1016/j.amjcard.2012.08.018 23000103 PMC3511626

[37] Lisitsa A Moshkovskii S Chernobrovkin A Ponomarenko E Archakov A. Profiling proteoforms: promising follow-up of proteomics for biomarker discovery. Expert Rev Proteomics (2014) 11(1):121–9. 10.1586/14789450.2014.878652 24437377

[38] Morris KV Mattick JS. The rise of regulatory RNA. Nat Rev Genet (2014) 15(6):423–37. 10.1038/nrg3722 24776770 PMC4314111

[39] Barnes EA Kenerson HL Jiang X Yeung RS. Tuberin regulates E-cadherin localization: implications in epithelial-mesenchymal transition. Am J Pathol (2010) 177(4):1765–78. 10.2353/ajpath.2010.090233 20813961 PMC2947273

[40] Sproul AA Vensand LB Dusenberry CR Jacob S Vonsattel JP Paull DJ Generation of iPSC lines from archived non-cryoprotected biobanked dura mater. Acta Neuropathol Commun (2014) 2(1):4. 10.1186/2051-5960-2-4 24398250 PMC3895779

[41] Gaskell G Allansdottir A Allum N Castro P Esmer Y Fischler C The 2010 Eurobarometer on the life sciences. Nat Biotechnol (2011) 29(2):113–4. 10.1038/nbt.1771 21301431

[42] Gaskell G Gottweis H Biobanks need publicity. Nature (2011) 471(7337):159–60. 10.1038/471159a 21390108

[43] Boycott KM Vanstone MR Bulman DE MacKenzie AE. Rare-disease genetics in the era of next-generation sequencing: discovery to translation. Nat Rev Genet (2013) 14(10):681–91. 10.1038/nrg3555 23999272

[44] Bell CJ Dinwiddie DL Miller NA Hateley SL Ganusova EE Mudge J Carrier testing for severe childhood recessive diseases by next-generation sequencing. Sci Transl Med (2011) 3(65):65ra4. 10.1126/scitranslmed.3001756 21228398 PMC3740116

[45] Davey Smith G Ebrahim S Lewis S Hansell AL Palmer LJ Burton PR. Genetic epidemiology and public health: hope, hype, and future prospects. Lancet (2005) 366(9495):1484–98. 10.1016/S0140-6736(05)67601-5 16243094

[46] Hewitt R Hainaut P. Biobanking in a fast moving world: an international perspective. J Natl Cancer Inst Monogr (2011) 2011(42):50–1. 10.1093/jncimonographs/lgr005 21672898

